# Role of community engagement in maternal health in rural Pakistan: Findings from the CLIP randomized trial

**DOI:** 10.7189/jogh.11.04045

**Published:** 2021-07-17

**Authors:** Zahra Hoodbhoy, Sana Sadiq Sheikh, Rahat Qureshi, Javed Memon, Farrukh Raza, Mai-Lei Woo Kinshella, Jeffrey N Bone, Marianne Vidler, Sumedha Sharma, Beth A Payne, Laura A Magee, Peter von Dadelszen, Zulfiqar A Bhutta, the CLIP Pakistan Working Group*, Amjad Hussain, Javed Memon, Farrukh Raza, Sharla K Drebit, Chirag Kariya, Mansun Lui, Diane Sawchuck, Ugochi V Ukah, Mai-Lei Woo Kinshella, Shafik Dharamsi, Guy A Dumont, Tabassum Firoz, Ana Pilar Betrán, Susheela M Engelbrecht, Veronique Filippi, William A Grobman, Marian Knight, Ana Langer, Simon A Lewin, Gwyneth Lewis, Craig Mitton, Nadine Schuurman, James G Thornton, France Donnay, Kelly Pickerill

**Affiliations:** 1 Department of Pediatrics and Child Health, The Aga Khan University, Karachi, Pakistan; 2 Department of Obstetrics and Gynecology, The Aga Khan University, Karachi, Pakistan; 3 Department of Obstetrics & Gynaecology, University of British Columbia, Vancouver, BC, Canada; 4 Department of Women and Children’s Health, School of Life Course Sciences, Faculty of Life Sciences and Medicine, King’s College London, St. Thomas’ Hospital, London, UK; 5 Center of Excellence in Women & Child Health, The Aga Khan University, Pakistan and East Africa, Karachi, Pakistan; 6 Centre for Global Child Health, Hospital for Sick Children, Toronto, Ontario, Canada

## Abstract

**Background:**

Community-based strategies to promote maternal health can help raise awareness of pregnancy danger signs and preparations for emergencies. The objective of this study was to assess change in birth preparedness and complication readiness (BPCR) and pregnant women’s knowledge about pre-eclampsia as part of community engagement (CE) activities in rural Pakistan during the Community Level Interventions for Pre-eclampsia (CLIP) Trial.

**Methods:**

The CLIP Trial was a cluster randomized controlled trial that aimed to reduce maternal and perinatal morbidity and mortality using CE strategies alongside mobile health-supported care by community health care providers. CE activities engaged pregnant women at their homes and male stakeholders through village meetings in Hyderabad and Matiari in Sindh, Pakistan. These sessions covered pregnancy complications, particularly pre-eclampsia/eclampsia, BPCR and details of the CLIP intervention package. BPCR was assessed using questions related to transport arrangement, permission for care, emergency funds, and choice of facility birth attendant for delivery during quarterly household surveys. Outcomes were assessed via multilevel logistic regression with adjustment for relevant confounders with effects summarized as odds ratios and 95% confidence intervals.

**Results:**

There were 15 137 home-based CE sessions with pregnant women and families (n = 46 614) and 695 village meetings with male stakeholders (n = 7784) over two years. The composite outcomes for BPCR and pre-eclampsia knowledge did not differ significantly between trial arms. However, CE activities were associated with improved pre-eclampsia knowledge in some areas. Specifically, pregnant women in the intervention clusters were twice as likely to know that seizures could be a complication of pregnancy (odds ratio (OR) = 2.17, 95% confidence interval (CI)  = 1.11, 4.23) and 2.5 times more likely to know that high blood pressure is potentially life-threatening during pregnancy (OR = 2.52, 95% CI = 1.31, 4.83) vs control clusters.

**Conclusions:**

The findings suggested that a CE strategy for male and female community stakeholders increased some measures of knowledge regarding complications of pre-eclampsia in low-resource settings. However, the effect of this intervention on long-term health outcomes needs further study.

**Trial registration:**

Clinical Trials.gov – INCT01911494.

The 1978 Alma Ata Declaration highlighted the pivotal role of individuals and communities in improving primary health care [1]. Despite several favorable large-scale community-based health projects in low-resource settings, these studies have largely failed to significantly improve maternal and child health [2]. The inability to involve communities to address and sustain engagement on these health issues after projects are completed may explain the lack of progress on these indicators [2]. It is hence that the World Health Organization’s Global Strategy for Women’s, Children’s and Adolescents’ Health (2016-2030) emphasizes community engagement (CE) to ensure the eventual goal of women and children not only surviving but also thriving [3].

CE is defined as the process of working collaboratively with people affiliated by geographic proximity, special interest, or similar situations to address issues affecting their well-being and ensures community participation in social change [4]. Previous maternal and child health projects involving CE includes the pioneering Warmi Project in rural Bolivia (1990-1993) that successfully involved community members to reduce perinatal mortality [5]. Similar projects were conducted in Nepal and Pakistan where health education with CE, using support groups, significantly improved neonatal health [6,7]. A recent meta-analysis on CE focusing on the effects of birth preparedness and provision of safe delivery kits reported a 60% lower risk of early neonatal mortality [8]. These projects demonstrate that systematic involvement of communities has potential to improve maternal and child health in low-resource settings [9] by facilitating change in socio-environmental risk factors, increased female empowerment due to greater decision-making power and promoting health-seeking behaviors [2].

CE strategies for maternal-newborn care include promotion of preventative newborn practices, the importance of antenatal care, knowledge of danger signs in pregnancy, and community action to address barriers to care such as transport and cost [10]. These strategies underpinned the CE activities of the Pakistan Community Level Intervention for Pre-eclampsia (CLIP) Trial [11]. The objective of this analysis was to evaluate the effect of CE activities on birth preparedness and complication readiness (BPCR) and pre-eclampsia knowledge of pregnant women in rural Sindh enrolled in the CLIP Pakistan Trial.

## METHODS

### Study design

The CLIP Pakistan Trial was one of three completed cluster randomized controlled trials conducted with pregnant women in Pakistan, India and Mozambique (2014-2016) (Clinical Trials.gov NCT01911494) [11-14]. The project was reported according to the CONSORT 2010 Statement (see Table S2 in the **Online Supplementary Document** for the checklist) [15]. The CLIP Pakistan Trial aimed to reduce all-cause maternal and perinatal morbidity and mortality through (a) CE, (b) a mobile health decision-aid to identify women at greatest risk, and (c) provide emergency care and referral by community health care providers [11,16].

### Study outcomes assessing the effects of community engagement

Effects of CE were assessed using BPCR and pre-eclampsia knowledge outcomes. The composite BPCR score was defined as at least two of the following: transport planning, obtaining permission for emergency care, money saved for emergency care, identified skilled birth attendant and health facility for delivery [17]. The composite pre-eclampsia knowledge score was defined as awareness of at least one pregnancy complications (abnormal bleeding after delivery, seizures, high blood pressure and life-threatening nature of high blood pressure during pregnancy) and at least four symptoms of hypertension in pregnancy.

### Community engagement participants and activities

In Pakistan, the definitive CLIP Trial was conducted in 20 clusters (10 in each arm) in Matiari and Hyderabad, Sindh Province. In the intervention clusters, CE activities were conducted at two levels: (1) male stakeholder sessions were conducted by a dedicated research team at community meeting places and (2) sessions with pregnant women and their families were held by government-deployed, female community health workers known as Lady Health Workers (LHWs) in the pregnant women’s homes using educational aides developed for the study [11]. Broadly adapted from the work by Lavery et al. [18], the principles of engagement included understanding local perceptions and building community trust, explaining the purpose of the current work, and continuous evaluation of the CE strategies.

**Figure 1:**
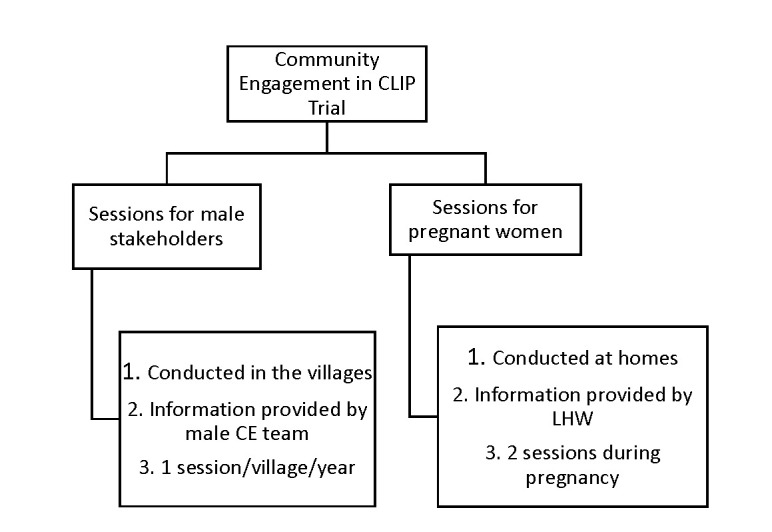
Community Engagement in the CLIP Trial. CLIP – Community Level Interventions for Pre-eclampsia, CE – community engagement, LHW – Lady Health Workers.

In the male stakeholder sessions, the CE team was responsible for contacting community stakeholders including landlords, social workers, political activists, religious leaders, and husbands. After mutual consensus of a scheduled date and venue, the CE team arranged an interactive 45-minute session in each village once per year (90 villages per cluster). Interactive sessions with pregnant women were conducted at first contact during pregnancy and subsequently during the third trimester at their homes with their families by LHWs using pictorial booklets developed for the trial. The content of messages delivered by LHWs were the same as those for male CE sessions (**Figure 1**).

The messages delivered during CE sessions included provision of antenatal care by a skilled health care provider, choice of provider and facility for delivery, danger signs during pregnancy, characteristics and frequency of pre-eclampsia/eclampsia, prior permissions in case of obstetric emergencies, transport plans, emergency funds for maternal and newborn complications and details of the CLIP intervention package. Women in control clusters received the standard of care, including routine LHW home visits where health promotion messages focused on antenatal care seeking, danger signs during pregnancy, skilled attendance at delivery, dietary advice and basic neonatal care.

### Statistical analysis

Maternal demographic and pregnancy characteristics were summarized between arms with medians and interquartile range (IQR) for continuous variables and counts and frequencies for categorical variables. BPCR and PE knowledge (both composite and components) were compared between arms with multi-level logistic regression models, with cluster as a random effect, adjusting for *a priori* specified variables thought to be possible predictors of the outcomes, which included: maternal age, maternal and husband’s education, nulliparity, gestational age at booking, and time (in months) from trial start date. Effects are summarized with odds ratios and 95% Wald type confidence intervals. Further, to assess possible lagged effects of the intervention, we carried out a second analysis with the above models for the composite outcomes, including an additional interaction between trial date and arm, which was tested for significance via the likelihood ratio test. All analyses excluded the pilot trial as CE activities were yet to be fully operational and include only women who delivered and had follow-up data at end of the trial. All analyses were conducted using R statistical software version 3.5.3 [19].

Ethical approval was obtained from the ethical review committees at The Aga Khan University Ethical (2590-Obs-ERC-13) and University of British Columbia (H12-03497).

Trial registration: Clinical Trials.gov, INCT01911494. Registered 30 July 2013, https://clinicaltrials.gov/ct2/show/NCT01911494.

## RESULTS

We recruited 16 766 women from the 10 intervention clusters and 15 829 women from the 10 control clusters (2015-2016). The sociodemographic and clinical characteristics of study participants were comparable between the two arms (**Table 1**).

**Table 1:** Sociodemographic and clinical characteristics of study participants.

	Intervention (N = 16 766)	Control (N = 15 829)
Age	28.00 (25.00, 30.00)	28.00 (25.00, 30.00)
Pregnant women with primary education*	3304 (19.7%)	2779 (17.6%)
Husbands with primary education*	8005 (47.9%)	6934 (43.9%)
Parity	2.00 (1.00, 4.00)	2.00 (1.00, 4.00)
Gestational age at enrolment (weeks)	20.21 (14.50, 27.07)	21.06 (15.20, 27.64)
Gestational age at delivery (weeks)	38.71 (36.14, 41.00)	38.71 (35.85, 41.00)
**Time from start of trial to delivery (months):**		
≤6	3732 (22.3%)	3430 (21.7%)
6-12	4679 (27.9%)	4451 (28.1%)
12-18	4341 (25.9%)	4180 (26.4%)
18-24	4014 (23.9%)	3768 (23.8%)
**Labour and delivery:**		
Facility birth	12 468 (76.7%)	11 682 (75.9%)
Skilled birth attendant at delivery	14 584 (87.0%)	14 162 (89.5%)
Cesarean section	2982 (17.8%)	2538 (16.1%)

*Primary education is equivalent to 5 years.

The male stakeholder CE sessions (n = 695) included 7784 participants including village heads, political leaders, social workers, schoolteachers/ principals, religious leaders, doctors and landlords while home-based LHW sessions (n = 15 137) included 46 614 pregnant women, husbands and their extended families.

**Table 2** reports the BPCR and pre-eclampsia knowledge outcomes. Pregnant women in the intervention clusters were twice as likely to state seizures as a complication of pregnancy as compared to control clusters (OR = 2.17, 95% CI = 1.11, 4.23). The intervention cluster participants were 2.5 times more likely to state high blood pressure as life-threatening during pregnancy as compared to controls (OR = 2.52, 95% CI = 1.31,4.83). However, the composite outcome for PE knowledge (OR = 1.74, 95% CI = 0.64, 4.73) and none of the BPCR individual components significantly differed between the two groups.

**Table 2:** Birth preparedness and complication readiness and pre-eclampsia knowledge

	Intervention (N = 16 766)	Control (N = 15 829)	Odds ratio (95% CI)	*P-* value
**Birth preparedness:**				
Composite*	7351 (43.87%)	4703 (29.72%)	1.74 (0.64, 4.73)	0.278
Arranged transport	6951 (41.49%)	3461 (21.87%)	2.77 (0.70, 10.96)	0.146
Has permission for antenatal care	14870 (98.4%)	14562 (96.2%)	0.45 (0.19, 1.06)	0.067
Has permission for emergency care	15 632 (94.81%)	15 027 (96.36%)	0.59 (0.22, 1.59)	0.297
Has money saved for emergency	4816 (28.74%)	3952 (24.98%)	1.21 (0.58, 2.52)	0.605
Identified facility	11 920 (75.17%)	10 922 (73.13%)	1.00 (0.62, 1.62)	0.989
Identified skill birth attendant	1823 (38.75%)	1637 (33.82%)	1.38 (0.53, 3.62)	0.514
**Pre-eclampsia knowledge:**				
Composite*	2785 (16.62%)	2301 (14.54%)	1.13 (0.49, 2.63)	0.769
Aware that women can have abnormal bleeding after delivery	12 673 (75.63%)	9522 (60.19%)	2.21 (0.77, 6.36)	0.140
Aware that women can have seizures during pregnancy	7607 (45.4%)	4560 (28.82%)	2.17 (1.11, 4.23)	0.023
Are you aware that women can have high blood pressure during pregnancy	10 668 (63.67%)	7771 (49.11%)	2.13 (0.97, 4.65)	0.058
Aware high blood pressure can be life threatening during pregnancy	8371 (49.96%)	4741 (29.96%)	2.52 (1.31, 4.83)	0.005
Identify four symptoms of high-blood pressure in pregnancy	3064 (18.29%)	2871 (18.14%)	1.1 (0.47, 2.59)	0.819

CI – confidence interval

*Adjusted for: cluster (random effect), maternal age, maternal primary education, husband primary education, parity, gestational age at enrolment, and months from start of definitive trial to delivery.

**Figure 2:**
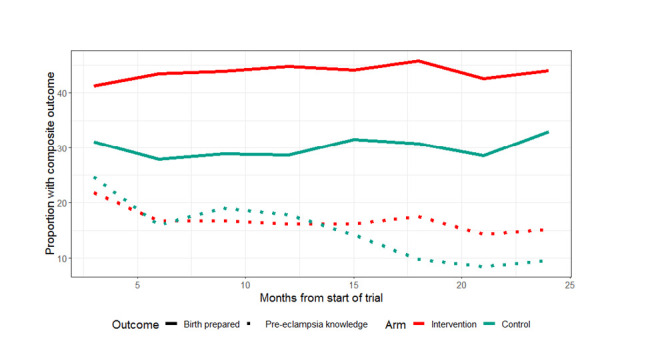
Birth preparedness and pre-eclampsia knowledge over the duration of the trial.

**Figure 3:**
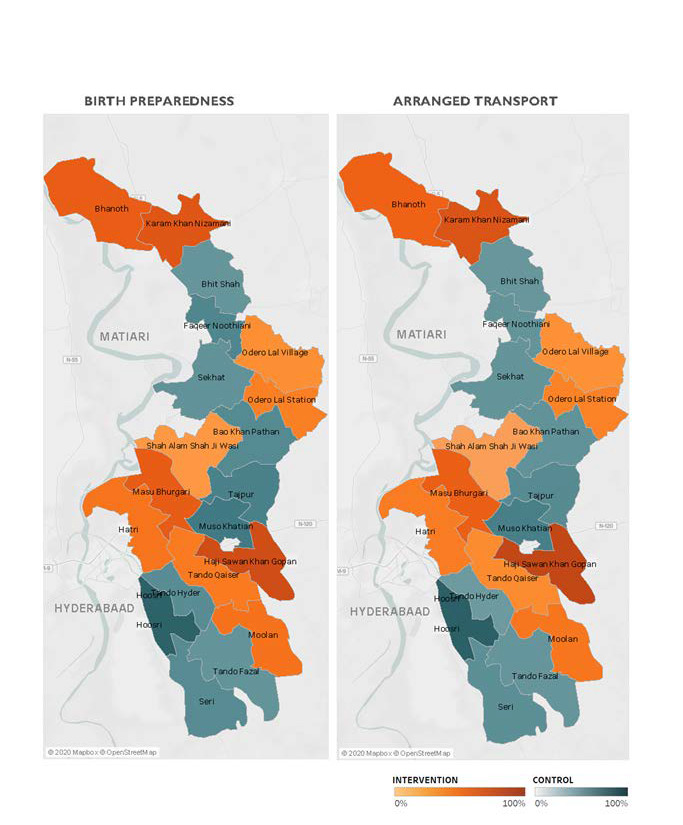
Composite birth preparedness and arranged transport by cluster.

**Figure 2** illustrates BPCR and pre-eclampsia knowledge over the duration of the trial. BPCR outcomes did not show much variation over the trial period while pre-eclampsia knowledge showed a statistically significant difference in trend over time between intervention and control clusters (*P* < 0.001). Details are presented in Figure S1 in the **Online Supplementary Document**. **Figure 3** illustrates a map of the BPCR composite outcome and those who had reportedly arranged for transport by cluster. Rates ranged from 90.1% (Hoosri) to 11.4% (Bhit Shah) of women meeting the BPCR composite threshold. While intervention clusters had higher rates of women who had arranged for transport than women in control clusters (41.5% vs 21.9%, **Table 2**), the map illustrates large variations between clusters within each trial arm.

## DISCUSSION

The CLIP Trial successfully demonstrated that engagement of male and female community stakeholders with the proposed strategy is a practical model to have an impact on pre-eclampsia knowledge in low-resource settings. However, as implemented, this did not translate into a difference in birth preparedness indicators.

The World Health Organization has identified CE interventions as one of the key pillars to empower communities to improve the health and well-being of women and children [20]. The poor health literacy in Pakistan is a potential contributor to the high burden of maternal and neonatal morbidity and mortality [21]. Previous efforts have demonstrated improved knowledge and health seeking behaviors for child health using CE strategies by community health workers in Pakistan [22,23]. However, evidence for the effect of CE activities on maternal health is lacking. Women in these communities previously reported a lack of awareness and misperceptions regarding the causes (eg, maternal stress), signs and symptoms (feeling weak), and the effect of pre-eclampsia on the mother and the newborn [24]. Similar findings have been reported from India where pregnant women have shown limited understanding of pre-eclampsia and its complications [25]. The CE sessions appear to lead to increased knowledge regarding the complications of pre-eclampsia (seizures and mortality), it did not have a significant effect on the identification of symptoms. Improvements in knowledge of danger signs of pregnancy and childbirth have been reported using a home-based CE strategy in rural Tanzania [26]. Despite the change in knowledge regarding causes and symptomatology of disease, translation into behavior change may need longer and sustained efforts as they may be deeply rooted in traditional beliefs and practices [24].

Despite a beneficial effect on neonatal mortality, recent systematic reviews have reported an inconclusive association of BPCR on knowledge regarding maternal danger signs [27] and skilled attendants at delivery [28]. The effects were most promising when at least 30% of women received the targeted intervention at home visits or through CE strategies [27]. Factors such as women’s education or features such as receipt of antenatal care in the last pregnancy and history of stillbirth are strongly associated with BPCR [29]. The CE approach adopted in the CLIP Pakistan Trial did not result in a statistically significant improvement in BPCR outcomes. This may be due to low (<30%) coverage of LHWs in certain areas, as was noted in the primary trial findings. In the CLIP Pakistan Trial the a benefit in clinical outcomes was limited to women in the intervention arm who received at least four home-based visits [11]; in the three-trial individual participant data meta-analysis, this was observed for those women who received at least eight visits [12].

Other plausible reasons for the lack of this difference may include poor literacy among pregnant women, financial constraints, community factors such as perceptions about BPCR and health systems factors such as distance from or prior experience at the health facility [30]. It has been demonstrated that the status of women in the community, employment status and autonomy for decision making are key drivers for maternal care seeking [31]. These require systemic action on individual, social and economic dimensions of role of women in the community [32]. CE may help increase health-related knowledge, but cultural and environmental changes on these dimensions may require a multi-pronged approach to address inequities and translate into behavior change.

The final explanation for the lack of between-arm difference in BPCR may be the impact of the three-monthly household surveillance during which the data for pre-eclampsia awareness and BPCR analyses were obtained. Questioning women and their family members about their knowledge of pre-eclampsia and whether or not they had prepared for obstetric emergencies may have sensitized families and had an indirect effect on their planning. This might explain the parallel temporal trends to increased transport planning in both trial arms (Figure S1 in the **Online Supplementary Document**).

The CE strategy for the CLIP Trial was designed for the specific cultural context of Sindh Province, Pakistan, and to overcome barriers observed in the prior feasibility assessment [24,33,34]. CE sessions were planned and delivered in a manner that was feasible according to the communities’ values and attitudes. This was reflected in the preparation, conduct and delivery of separate sessions for me and pregnant women. The strategy of addressing men at common gathering places, regardless of age, marital status or their wife’s pregnancy status and the presence of their peers helped to disseminate the messages throughout the broader community. Furthermore, conducting CE sessions with pregnant women in their homes and in the presence of their female relatives and friends increased acceptability. Effective community partnerships need to be built upon trust, shared vision for improving the health of the community, and pooling of physical and social capital resources [35]. The CE sessions were a medium to build rapport, impart knowledge regarding pregnancy complications in general and pre-eclampsia specifically and to emphasize the importance of birth preparedness. LHWs are an existing health workforce; generally, LHWs are residents of the area and have a mandate to deliver preventive and promotive health messages in the community [36]. Therefore, this cadre was ideal to deliver CE messages to pregnant women, both for acceptability from the community and sustainability of the process. Even though it was not studied independently, male involvement was ensured through separate CE sessions in the CLIP Pakistan Trial as they have shown to improve care-seeking and birth preparedness outcomes [37]. Male involvement in maternal health has shown to have a positive impact on antenatal care seeking and skilled birth attendance in rural Ethiopian women [38].

The CLIP Pakistan Trial included a large randomized cohort of pregnant women in rural Pakistan to assess the effects of the intervention on maternal and neonatal morbidity and mortality. This was one of the few systematic efforts to engage male stakeholders from a rural Pakistani community in BPCR and health promotion regarding pregnancy. However, there are a few limitations to this study. The timing of the CE session and knowledge assessment during the quarterly surveillance could range from days up to three months postpartum and may have an influence on the findings. The BPCR and pre-eclampsia composite outcomes have not been validated and may not represent the desired markers for assessment of these parameters (as indicated by the individual pre-eclampsia knowledge outcomes); these issues may limit their interpretation. This also highlights the need for a validated tool to measure BPCR in a robust manner in low-resourced settings. The impact of CE activities has been difficult to measure due to the lack of reliable and standardized metrics and hence has mainly focused on process evaluation [39].

Due to the nature of the intervention in the CLIP Pakistan Trial, it was difficult to tease out the effect of a single intervention component (ie, change in knowledge and behaviors advocated through CE sessions) on maternal care seeking or morbidity and mortality.

## CONCLUSION

While the CE strategies adopted in the CLIP Pakistan Trial helped improve knowledge regarding complications of pre-eclampsia, they did not have an impact on overall birth preparedness in this low-resource setting. However, the effect of this increase in knowledge on health outcomes needs to be further assessed. Further research is required to assess the impact of a contextual CE model on knowledge and behaviors of men and women regarding pregnancy and childbirth to improve maternal and neonatal health in these communities.

## Additional material

Online Supplementary Document
