# Supplementary material for: Role of community engagement in maternal health in rural Pakistan: Findings from the CLIP randomized trial
Source: J Glob Health. 2021 Jul 17;11:04045. doi: 10.7189/jogh.11.04045 (PMC8285765; doi:10.7189/jogh.11.04045)
Supplement: Online Supplementary Document [file jogh-11-04045-s001.pdf]

Online Supplementary Document

**Table S1:** CLIP Pakistan Working Group	Page 2
**Table S2:** CONSORT 2010 checklist of information to include when reporting a randomised trial	Page 3
**Figure S1:** Individual component of pre-eclampsia awareness (left) and birth preparedness and complication readiness (right) over time	Page 6

Table S1: CLIP Pakistan Working Group.

First and middle names	Last names
Amjad	Hussain
Javed	Memon
Farrukh	Raza
Sharla K	Drebit
Chirag	Kariya
Mansun	Lui
Diane	Sawchuck
Ugochi V	Ukah
Mai-Lei Woo	Kinshella
Shafik	Dharamsi
Guy A	Dumont
Tabassum	Firoz
Ana Pilar	Betrán
Susheela M	Engelbrecht
Veronique	Filippi
William A	Grobman
Marian	Knight
Ana	Langer
Simon A	Lewin
Gwyneth	Lewis
Craig	Mitton
Nadine	Schuurman
James G	Thornton
France	Donnay
Kelly	Pickerill

Table S2: CONSORT 2010 checklist of information to include when reporting a randomised trial.

Section/Topic	Item No	Checklist item	Reported on page No
**Title and abstract**			
	1a	Identification as a randomised trial in the title	Page 1
	1b	Structured summary of trial design, methods, results, and conclusions (for specific guidance see CONSORT for abstracts)	Page 2-4
**Introduction**			
Background and objectives	2a	Scientific background and explanation of rationale	Page 4-5
	2b	Specific objectives or hypotheses	Page 5
**Methods**			
Trial design	3a	Description of trial design (such as parallel, factorial) including allocation ratio	Page 5
	3b	Important changes to methods after trial commencement (such as eligibility criteria), with reasons	N/A*
Participants	4a	Eligibility criteria for participants	Page 6
	4b	Settings and locations where the data were collected	Page 6
Interventions	5	The interventions for each group with sufficient details to allow replication, including how and when they were actually administered	Page 6-7
Outcomes	6a	Completely defined pre-specified primary and secondary outcome measures, including how and when they were assessed	Page 5-6
	6b	Any changes to trial outcomes after the trial commenced, with reasons	N/A*
Sample size	7a	How sample size was determined	N/A*
	7b	When applicable, explanation of any interim analyses and stopping guidelines	N/A*
Randomisation:			

Sequence generation	8a	Method used to generate the random allocation sequence	N/A*
	8b	Type of randomisation; details of any restriction (such as blocking and block size)	N/A*
Allocation concealment mechanism	9	Mechanism used to implement the random allocation sequence (such as sequentially numbered containers), describing any steps taken to conceal the sequence until interventions were assigned	N/A*
Implementation	10	Who generated the random allocation sequence, who enrolled participants, and who assigned participants to interventions	N/A*
Blinding	11a	If done, who was blinded after assignment to interventions (for example, participants, care providers, those assessing outcomes) and how	N/A*
	11b	If relevant, description of the similarity of interventions	N/A*
Statistical methods	12a	Statistical methods used to compare groups for primary and secondary outcomes	Page 7
	12b	Methods for additional analyses, such as subgroup analyses and adjusted analyses	Page 7
**Results**			
Participant flow (a diagram is strongly recommended)	13a	For each group, the numbers of participants who were randomly assigned, received intended treatment, and were analysed for the primary outcome	N/A*
	13b	For each group, losses and exclusions after randomisation, together with reasons	N/A*
Recruitment	14a	Dates defining the periods of recruitment and follow-up	Page 8
	14b	Why the trial ended or was stopped	N/A*
Baseline data	15	A table showing baseline demographic and clinical characteristics for each group	Page 8, table 1
Numbers analysed	16	For each group, number of participants (denominator) included in each analysis and whether the analysis was by original assigned groups	Page 8, table 1
Outcomes and estimation	17a	For each primary and secondary outcome, results for each group, and the estimated effect size and its precision (such as 95% confidence interval)	Page 9, table 2

	17b	For binary outcomes, presentation of both absolute and relative effect sizes is recommended	N/A*
Ancillary analyses	18	Results of any other analyses performed, including subgroup analyses and adjusted analyses, distinguishing pre-specified from exploratory	N/A*
Harms	19	All important harms or unintended effects in each group (for specific guidance see CONSORT for harms)	N/A*
**Discussion**			
Limitations	20	Trial limitations, addressing sources of potential bias, imprecision, and, if relevant, multiplicity of analyses	Page 13-14
Generalisability	21	Generalisability (external validity, applicability) of the trial findings	Page 13
Interpretation	22	Interpretation consistent with results, balancing benefits and harms, and considering other relevant evidence	Page 10-14
**Other information**			
Registration	23	Registration number and name of trial registry	Page 5
Protocol	24	Where the full trial protocol can be accessed, if available	Page 5
Funding	25	Sources of funding and other support (such as supply of drugs), role of funders	Page 15

*For more details on the methodology, implementation and results of the full CLIP Trial in Pakistan, please see: Qureshi RN, Sheikh S, Hoodbhoy Z, Sharma S, Vidler M, Payne BA, et al. Community-level interventions for pre-eclampsia (CLIP) in Pakistan: A cluster randomised controlled trial. Pregnancy Hypertens. 2020 Oct 1;22:109–18. doi: 10.1016/j.preghy.2020.07.011.

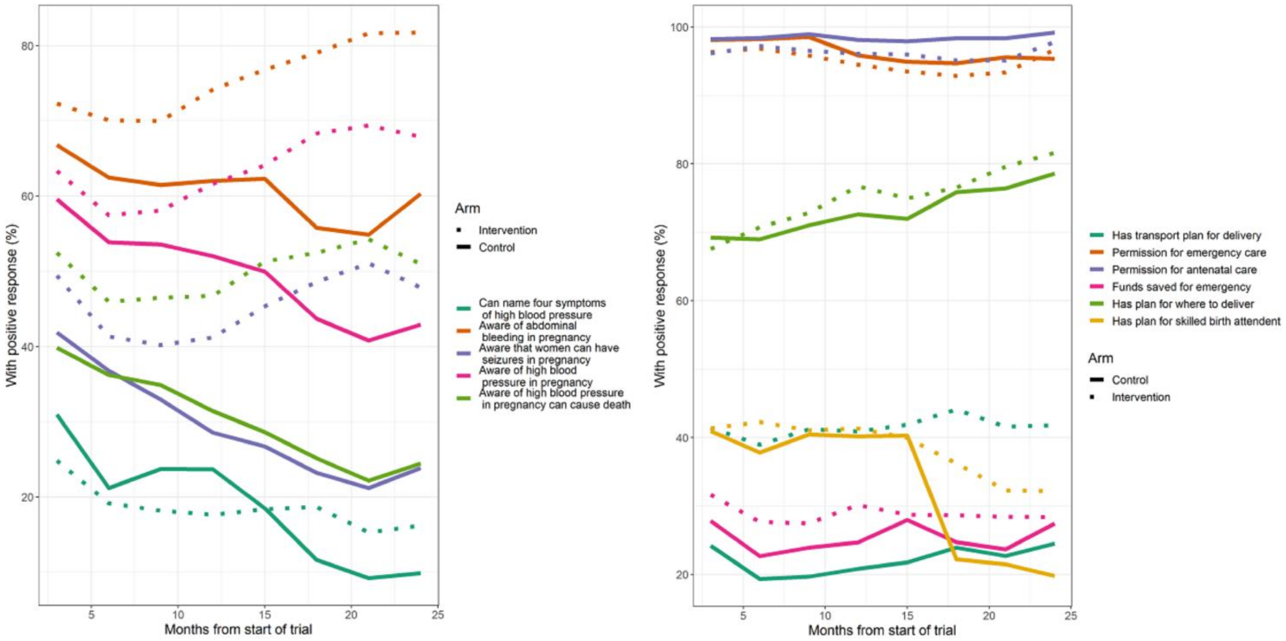

Figure S1: Individual component of pre-eclampsia awareness (left) and birth preparedness and complication readiness (right) over time.
